# Community mobilization for malaria elimination: application of an open space methodology in Ruhuha sector, Rwanda

**DOI:** 10.1186/1475-2875-13-167

**Published:** 2014-05-02

**Authors:** Chantal Marie Ingabire, Jane Alaii, Emmanuel Hakizimana, Fredrick Kateera, Daniel Muhimuzi, Ingmar Nieuwold, Karsten Bezooijen, Stephen Rulisa, Nadine Kaligirwa, Claude Muvunyi, Constantianus JM Koenraadt, Leon Mutesa, Michele Van Vugt, Bart Van Den Borne

**Affiliations:** 1 Department of Health Promotion, Maastricht University, Maastricht, The Netherlands; 2 Medical Research Center, Rwanda Biomedical Center, Kigali, Rwanda; 3 Context-Factor Solutions, Nairobi, Kenya; 4 Malaria & Other Parasitic Diseases Division, Rwanda Biomedical Center, Kigali, Rwanda; 5 Academic Medical Center, Amsterdam, The Netherlands; 6 Amasezerano Community Banking, Kigali, Rwanda; 7 Foundation The100th Village, Amsterdam, The Netherlands; 8 Department of Gynecology, Kigali University Teaching Hospital, Kigali, Rwanda; 9 College of Medicine and Health Sciences, University of Rwanda, Kigali, Rwanda; 10 National Reference Laboratory, Rwanda Biomedical Center, Kigali, Rwanda; 11 Wageningen University, Wageningen, The Netherlands

**Keywords:** Malaria, Participatory approaches, Community engagement, Rwanda

## Abstract

**Background:**

Despite the significant reduction of malaria transmission in Rwanda, Ruhuha sector is still a highly endemic area for malaria. The objective of this activity was to explore and brainstorm the potential roles of various community stakeholders in malaria elimination.

**Methods:**

Horizontal participatory approaches such as ‘open space’ have been deployed to explore local priorities, stimulate community contribution to project planning, and to promote local capacity to manage programmes. Two open space meetings were conducted with 62 and 82 participants in years 1 and 2, respectively. Participants included purposively selected community and local organizations’ representatives.

**Results:**

Malaria was perceived as a health concern by the respondents despite the reported reduction in prevalence from 60 to 20% for cases at the local health centre. Some misconceptions of the cause of malaria and misuse of preventive strategies were noted. Poverty was deemed to be a contributing factor to malaria transmission, with suggestions that improvement of living conditions for poor families might help malaria reduction. Participants expressed willingness to contribute to malaria elimination and underscored the need for constant education, sensitization and mobilization towards malaria control in general. Active diagnosis, preventative strategies and prompt treatment of malaria cases were all mentioned by participants as ways to reduce malaria. Participants suggested that partnership of stakeholders at various levels could speed up programme activities. A community rewards system was deemed important to motivate engaged participants, i.e., community health workers and households. Establishment of malaria clubs in schools settings was also suggested as crucial to speed up community awareness and increase skills towards further malaria reduction.

**Conclusions:**

This bottom-up approach was found useful in engaging the local community, enabling them to explore issues related to malaria in the area and suggest solutions for sustainable malaria elimination gains.

## Background

Malaria has received much attention over the years due to its impact on public health. There has been a significant increase in malaria cases worldwide, estimated at 225 million in 2009. Malaria is one of the leading causes of death in developing countries with 781,000 estimated annual deaths worldwide, 91% of which occur in Africa [1]. Many efforts have been put in place to control malaria. WHO recommends vector control, including individual personal protection against mosquito bites as a powerful primary public health intervention for reducing malaria transmission at community level, with reinforcement through behaviour change communication [2]. In Rwanda, impressive outcomes have been achieved in malaria control over recent years. Malaria mortality dropped to 16% of hospital deaths and 12% of outpatient consultations in 2008 compared to 41 and 37% of hospital deaths and outpatient consultations, respectively in 2006. The achievement is attributed firstly to widespread community participation of 90% in the national health insurance (*Mutuelles de santé*) scheme, and secondly to the use of insecticide-treated bed nets, indoor residual spraying, as well as the goal of the national malaria programme to target the entire population with malaria prevention and control measures [3].

Successful disease control at community level needs to take human behavior, sociocultural and economic context into account in parallel with biomedical interventions [4]. Engagement and participation of communities in planning, implementing and evaluating a control programme helps to ensure that a programme resonates with issues important to them and that findings are locally relevant [5].

Community participation is defined as a method of people working together through community structures in order to raise awareness and identify local ideas, concerns, priorities, and opportunities so as to enable them to achieve sustained provision of appropriate services [6]. Two conceptual approaches to community participation have been identified: vertical and horizontal. Vertical participation implies a centralized development of research objectives by policymakers with responsibility to engage the community, whereas horizontal participation entails facilitating communities to identify and define problems from their perspective and subsequently to help tailor solutions to specific context and needs. The horizontal approach is known to engender sustainability of community-oriented programmes through self-efficacy, social identity and empowerment [6,7]. Various horizontal participatory methods are known and have been deployed differently. For instance, while the information in Rural Rapid Appraisal (RRA) is driven more by outsiders as part of a process of data gathering, the locus of control in Participatory Rural Appraisal (PRA) – an advanced form of RRA – is attributed more to the participants [8]. On the other hand, Participatory Action Research (PAR) is mainly used in health related research and the method is based on reflection, data collection, and action that aim to improve health and reduce health inequities through involving the people who, in turn, take actions to improve their own health [9]. There is a large evidence-base where horizontal approaches have been successful due to a strong partnership between community and programme implementers. The key elements of these programmes are: 1) generation of a feeling of empowerment; 2) local ownership and responsibility; and, 3) the application of action-oriented and participatory approaches [10].

As part of multidisciplinary horizontal research, involving medical, entomological, economical as well as behavioural components with the aim to empower communities towards malaria elimination, the ‘open space’ as another participatory approach was deployed [11]. The method aims to explore and brainstorm community perceptions of the malaria problem in Rwanda-Ruhuha sector, their willingness to participate in a malaria elimination programme and to determine the key actions to be taken by various stakeholders. Similar to other horizontal participatory approaches, such as the participatory rural appraisal and participatory learning and action [12,13], the standard method of open space aims at facilitating participants to identify and explore issues that are important to them with regard to an identified problem, and to identify opportunities for change and set priorities among action steps to achieve desired goals in an innovative and productive way. Open Space engenders visualization through the use of cards on which participants write or draw illustrations. It also promotes an active participation process including facilitation and participation in small and large group sessions. Participants in open space are considered to be synergistic and self-motivated with an overarching assumption that those who respond to invitations are more concerned with the problem to be studied [14]. The method has not yet been applied in a public health context. Rather, it has been used in organizational change and is based on four principles: whoever comes is the right person; whatever happens is the only thing that could have; whenever it starts is the right time; and, when it is over, it is over. Uncommon in other participatory approaches, the law of mobility is a key element during open space. It allows participants to move to a more productive place if they are neither learning nor contributing to a certain group [15]. It is critical to explain to participants these principles, as well as the law of mobility, before group discussions are initiated. Two open space meetings were conducted at two different times among the Ruhuha community, focusing on two related calling questions: 1) as a community in what ways and how are you going to contribute to malaria reduction? and 2) what can we do as stakeholders to eliminate malaria in Ruhuha sector?

## Methods

### Study area, target population and sampling

Ruhuha sector is located in the southern province of the Republic of Rwanda. It occupies an area of 54 square kilometres with a population of 19,606. Ruhuha sector has about 5,000 households living in five cells, with 35 small villages in total. The sector is bordered by lake Cyohoha and has numerous marshlands and water streams. Wetland agriculture and rice cultivation is common practice. Lake Cyohoha is a major corridor for uncontrolled population movements between Rwanda and Burundi. Due to the high malaria incidence previously reported in Ruhuha sector and the long term working relationship with the health centre, the site was chosen to host the main study aiming at malaria elimination in which open space is embedded as one of the community participatory approaches to be deployed. The study population comprised a cross-section of selected male and female community representatives, including community health workers, health care professionals, school teachers, local NGO representatives, religious and local administration leaders, as well as members of the lay community.

The sample for participation was drawn from Ruhuha in collaboration with the Ruhuha health centre and the local administration of Ruhuha sector. Maximum variation sampling was used to enable identification of shared experiences across individuals representing a wide variation in dimensions of interest [16]. The majority of participants in the first open space also participated in the second open space as there were no changes in their roles as community representatives and also because the second open space was a follow-up of the previous one.

### Open space workshops

#### Overview

Open space, a way to constantly engage with, learn with and provide feedback to the community, is scheduled to be conducted each year in the project for a period of three years. This is prior to and after annual household surveys that are planned in the main study. Two open space sessions were conducted subsequently in year one and two. The first open space workshop was conducted in February 2010, followed by the second in June 2011 with 62 and 82 participants, respectively. The approach was primarily chosen to be used in this project due to the fact that it was seen as innovation to be used in public health settings while it was previously mainly used in organizational settings. Secondly, the approach adequately houses the critical qualities of participatory approaches without necessarily being resource intensive: (1) problem identification, (2) developing actions to remediate the problem(s), (3) taking mutually agreed actions, (4) evaluating the learning outcomes, and (5) reflecting and re-planning. In addition to that is the unique principle or power of mobility in open space. This enriches the diversity of contributions as members move around smaller group sessions – something that may be somewhat limited during open feedback sessions when the group is larger and shy participants may become too self conscious and less inclined to speak up.

Prior to the conduct of each open space, a planning meeting was held by the research team and selected community representatives to prepare the actual conduct of the discussions. At the same meeting, members discussed and agreed a calling question to be put on the community invitation letters. In collaboration with the Ruhuha health centre, a meeting room was made available to host the discussions. On the day of each open space workshop, team introductions and community representation were done. Explanations of open space technique and its principles were provided. Illiterate participants were explained that they may ask their group members to help write down their ideas. Free listing of participants’ expectations of the meeting has been done. From these listings, emerging themes and subthemes were noted for data synthesis. The third step involved brainstorming among participants with regard to the designed calling questions, specifically setting an agenda on what should be discussed in light of the calling question.

#### Calling questions

Bugesera district, specifically Ruhuha sector is highly malaria endemic. With this in mind, the first calling question designed focused primarily on malaria prevention and required actions towards its reduction, “*As a community in what ways and how are you going to contribute to malaria reduction?”* A synthesis of this data, and a follow-up meeting two days later with the health centre leadership and local administration suggested community willingness to participate in malaria reduction. Based on this insight and the cognizance that Rwanda is shifting to pre-elimination phase by 2017, the next calling question *“What can we do as stakeholders to eliminate malaria*? was designed. This was a follow-up of the previous session and focused on exploring the specific role of various stakeholders in malaria elimination.

#### Group work

Calling questions previously sent to the participants on the invitation letters were again repeated in an open plenary session. Emerging topics from those opening questions were regrouped into major themes to be discussed in small groups (see Results). Break-out groups were formed based on individual interests in topics resulting from the plenary discussion of the calling question. Participants were asked to first take part in their group of interest. Each group nominated a group facilitator together with a note taker. The standard law of mobility in the open space was applied, allowing participants to move to other groups once they felt they were no longer contributing or learning from their current group discussion. The implication is that mobile members only partly contribute in any given group, however; they enrich the discussion in other groups as well as being enriched by their participation in diverse groups. Groups ran concurrently and each group discussion session lasted about 45 minutes. Groups recorded notes on cards and a flipchart and presented in the closing plenary of the open space at the end of the session. During feedback, an opportunity was given to the participants to further elaborate any of their ideas, or clarify any idea they felt had not been captured in perspective. The opening and closing plenary sessions each took 15–20 minutes.

#### Data synthesis

Data synthesis following each open space meeting was done in two phases. Initial synthesis of emerging information was done with the community in the closing plenary. Note takers first presented the outcome of their group discussions followed by brief feedback or further input from the wider group. Finally, after all group presentations and feedback, participants were asked to evaluate whether their expectations had been met. Participants were asked to anonymously list their feedback on small index cards. The data synthesis team, comprising senior researchers and community participation facilitators, went through the listings made by participants. Data were first translated into English from Kinyarwanda and then categorized under key themes and subthemes that emerged from participants’ immediate expectations and feedback before and after open space, respectively. From group work, issues identified by participants in relation to malaria in the area, their proposed actions towards its elimination, the process towards achievement of the desired actions as well as the responsibility of stakeholders at each level were also qualitatively analyzed by category. Index cards bearing similar concerns/ideas were grouped together on board. This was followed with data interpretation meetings held with a small group of community opinion leaders (the head of the health centre where the open space was conducted, local administrative authorities, in charge of community health workers, community participatory specialists as well as the researchers) a day after each workshop.

#### Ethical considerations

The Rwanda National Ethics Committee approved the study protocol. Health and administrative authorities were informed prior to both open space meetings. The overall open space process was done in accordance to its principles, which were also well defined in advance to the participants. All discussions were conducted in the national language, *Kinyarwanda*, moderated by four PhD students, all fluent in the language and who had undergone training for conducting an open space workshop. Participant notification was done at least two weeks ahead of the workshops. Sessions were designed for open and free discussion where respondents were not obliged to respond to any question. Participants were reimbursed for their transportation costs.

## Results

### Characteristics of participants

Sixty-two (62) versus 82 participants took part in the two open space discussions. More men than women took part into the two open space (36 women versus 61 men respectively). The majority of participants in the first and second sessions were community health workers (58 and 44%, respectively). There was a marginal increase in the number of local authorities who attended the sessions in the second open space compared to the first (10 and 47.5%, respectively). There were fewer participants representing local NGOs who attended the second session compared to the first (13 *versus* 1%), mainly due to the end of activities for some NGOs in the area. A similar trend was noted among health care professionals who were present (8 *versus* 3.5%).

### Participants’ expectations of open space meetings

Themes resulting from participants’ expectations before and after open space are listed in descending order of the frequency with which they were listed. It is apparent from data that in both open space meetings the sample size (N) was lower than the total listings combined because each participant could list several expectations.

### Expectations before open space meetings

Participants’ expectations about the open space meeting are presented in Table 1. Many participants expressed a need for constant education and mobilization for malaria control in general, listed 57 and 55 times, respectively. This included understanding malaria transmission, prevention and treatment, and potential community roles for control and/or elimination. Participants further expressed their needs for specific education on the use of bed nets, indoor residual spraying and environmental management (e g, clearing mosquito breeding sites). The need for treatment literacy was emphasized by some participants, including education on when and where to seek health care, the prompt treatment of malaria and how malaria treatment works. Another element arising from participants’ expectations was the definition of different stakeholders’ roles. Participants emphasized that this was in order to make clear everyone’s responsibilities and to engage in partnership with each other to accelerate malaria elimination.

**Table 1:** Expectations of participants regarding the outcomes of open space meetings

**Stakeholder expectations and emerging themes before open space in 2010**		**Stakeholder expectations and emerging themes before open space in 2011**	
**Item**	**N = 62**	**Item**	**N = 82**
To be educated	57	To be educated	55
To mobilize the community	15	To understand roles of different stakeholders	10
To understand roles of different stakeholders	11	To mobilize the community	9
To have access to health services	5	To have access to health services	9
To ensure the follow-up of patients on treatment	2	To ensure the follow-up of patients on treatment	9
		Indoor residual spraying	7
		To distribute bed nets	5
		To clear the environment	4

Difficulties in seeking health care were mentioned in the two sessions. Participants mentioned the challenge in accessing health services, specifically in terms of unavailability of drugs to treat adults at community level, the long distance to the health centre and the issue of patient adherence to treatment, including completing the full dose prescribed and as instructed by health personnel.

### Expectations after open space meetings

Participants’ evaluation of the extent to which the open space meetings had met their expectations are presented in Table 2. Thirty-six versus 77 participants responded to the expectations evaluation posed after the first and second open space, respectively. Participants generally felt they had learned much from discussions in terms of malaria transmission, prevention and treatment. They also expressed commitment to providing feedback to their communities on what they had learned. This was more frequently mentioned in the second open space discussion than the first. Discussions also highlighted the perceptions of a critical need for supply of aids, such as educational flipcharts and skills training for community health workers to enable them to transfer the knowledge they gained to their respective communities. Participants in the second open space meeting seemed to view malaria elimination more from a community-oriented approach, identifying initiatives, implying the recognition of the malaria problem as their own and the need for them to be active contributors to actions to eliminate malaria. In contrast, the first open space suggested a ‘passive recipient’ approach characterized by the absence of participant recognition of their own capabilities and resources that could be put towards identified initiatives. Specifically, while participants in the first open space felt they had learned from the session, they appeared not to recognize the information to have been generated wholly from their discussions, instead ascribing it to the proposal development team, while at the same suggesting an inevitable need for external support towards funding, training, implementation, and a rewards system.

**Table 2:** Participant evaluations of open space outcomes

**Review of expectations after open space in 2010**		**Review of expectations after open space in 2011**	
**Item**	**N=36**	**Item**	**N=77**
Appreciation of participants to have gained more knowledge on malaria elimination	24	To exercise prevention measures	54
Willingness to act towards malaria elimination and sharing information to others	17	Willingness to act towards malaria elimination and sharing information to others	41
To understand the need of stakeholder partnership	10	Appreciation of participants to have gained more knowledge on malaria elimination	38
To establish a reward system for well recognized actions aiming at malaria elimination	6	To understand the need of stakeholder partnership	35
To provide infrastructural support and training (need materials to diagnose and treat malaria in adults by community health workers, sufficient bed net distribution, ongoing training by community health workers on innovation in malaria elimination)	5	Malaria elimination possible	12
		Importance of a health insurance	11
		To ensure targeted actions towards elimination	7
		Other	22

### Emerging themes from group discussions

Major themes emerging in the first open space included hygiene and cleanliness, self-protection against malaria either by using bed nets, indoor residual spraying (IRS) and integrated efforts of stakeholders, including community, donors and government. Themes from the second open space included improving the well-being of the Ruhuha population in order to be able to fight against malaria, referring to the reduction in poverty through participating in economical cooperatives, looking for special preventive strategies for vulnerable groups (children under five, pregnant women, persons living with HIV and tuberculosis), community ownership of the programme, active malaria diagnosis referring to household malaria diagnosis, environmental clearing by cutting bushes and removing mosquito breeding sites, full coverage by availability of mosquito nets for each bed and sleeping mat in the household, empowering community health workers towards diagnosis and treatment of adults through training and provision of diagnosis materials and medication.

### Community perceptions of malaria in Ruhuha

Malaria was recognized as a burden for the Ruhuha community, although the health centre reported a significant decrease from 60 to 20% of malaria cases at health centre level in 2010. They associated the decrease with community awareness of individual protection against mosquito bites by correct and regular use of bed nets, in addition to community-wide environmental cleanliness, including clearing of bush and other breeding sites, such as stagnant water. However, participants in both sessions reported some misuse of mosquito nets, for instance, for fishing and/or fencing off kitchen gardens. They also reported that misconceptions about malaria and its preventive measures still existed among the community, i.e., malaria being perceived as caused by eating sugar cane or due to personal hygiene, such as not taking shower, etc.

### Poverty reduction

It emerged that malaria is a problem of poverty, thus improving the livelihoods of poor families might help malaria elimination. Participants suggested this should be done through the promotion of agriculture and farming mechanisms that are best suited to the area, grouping the community into cooperatives to participate in sustainable, income-generating activities and thereby have access to savings. The latter was perceived to have the potential to contribute to the finances of those who were unable to pay for medical insurance, which resulted in delaying seeking health care or not seeking health care at all.

### Active diagnosis, prevention and treatment of malaria

Participants stated that availability of medicine for malaria prevention and a vaccine especially for vulnerable groups, such as children under five years of age, pregnant women, the elderly, HIV and tuberculosis patients, are important for malaria elimination. They mentioned that early diagnosis and treatment of malaria cases as well as follow-up with regard to adherence to treatment is vital. The work done by community health workers in treating children below the age of five years suffering from malaria appeared to be much more appreciated by the participants in the second open space, most likely due to the increase of absolute number compared to the first one. Therefore, they suggested that community health workers should be trained to be able to provide malaria diagnosis and treatment for adults as well. They felt this might have a positive impact on malaria elimination, because patients would be treated early and this would solve the issue of long distances to the health centre, time spent at the health centres, and generally significantly reduce the number of malaria cases that are received at health centre level.

With regard to vector control and environmental management, the emphasis was on prevention of mosquito bites by correct use of bed nets and on reducing mosquito reproduction through indoor residual spraying. However, participants raised concerns about the issue of fleas spreading in houses after spraying. This led them to suggest increasing the dose of sprays or making available sprays that can kill both mosquitoes and fleas. A few participants mentioned that sometimes spraying is done after the rice harvest which resulted in no efficacy because that it is the period when mosquito populations have reduced. They suggested that indoor residual spraying should be done on a regular basis, at the right season for optimum outcome. Several accounts stated that bed net coverage and their use is a major factor in malaria elimination, and the community participants suggested that they should be provided with enough bed nets to the number of beds in the house, that their distribution in boarding schools should be considered, and/or they should be available for retail at health centres or shops, at an affordable price. More effort in education focusing on correct bed net use was needed.

### Community mobilization, sensitization and education

Misconceptions about malaria preventative measures still existed among the local community, thus providing them with correct knowledge through sensitization, mobilization, training and education for local authorities, community health workers and community itself seemed to be an appropriate focus for malaria elimination. Participants expressed the need for repeated intensive education with a focus on malaria prevention methods, malaria symptoms, early diagnosis and treatment preferably at the hospital, correct use of bed nets, the benefits of indoor residual spraying, hygiene and environmental cleaning as well as the importance of having health insurance. Participants considered the involvement of various stakeholders as key to success in malaria elimination, including stakeholders at national level, community grassroots level, and partnership between the national and local level as well as other community organizations. It was proposed that central government be in charge of making available drugs and ongoing training for health professionals in Ruhuha. The health centre level was expected to organize local leaders and health workers, undertake monitoring and evaluation activities as well as to report back to central level. The community health workers were proposed to organize community meetings and conduct education and sensitization at community level and lastly the community to put into practice the preventive strategies as needed. The NGOs, churches, schools, and cooperatives were identified as other channels that could be used for community sensitization. Further, creation of malaria clubs was recommended to be used as the forum in which all malaria activities at community level are organized, coordinated and communicated to the wider community.

### A rewards system

Participants, particularly in the first open space meeting, highlighted a reward and incentive system as essential for maintaining motivation at different implementation levels because the reward system places attention on the importance of the health problem and participants recognized the community responsibility to contribute to the system. The rewards should assess firstly the village in which the level of engagement of its community members is excellent, secondly a community health worker who has done a lot in implementing and sensitizing others, and thirdly a household, which has significantly reduced malaria cases at their level.

## Discussion

Open space was used as a technique to be incorporated in the main integrated programme with the goal of malaria elimination in the community of Ruhuha-sector. The approach was deemed to serve as a platform where various community members were engaged in identifying factors promoting or hindering malaria elimination and look for feasible local solutions. The approach served as a way to assess community willingness to participate in malaria elimination. The observation that it served as an intervention strategy whereby participants felt they learnt a lot from being part of the discussions was an unexpected outcome. However, this is common with participatory approaches, attributable to the inherent reflective pattern of engagement in group settings.

The findings suggest that some misconceptions and misuse of preventive tools are still observed. This can hinder the achievement of malaria elimination and should be tackled to ensure there is no resurgence of malaria cases, reduced significantly at the level of health centre. This is in consistent with Roll Back Malaria [17], where community knowledge through constant mobilization, sensitization and education towards malaria preventative actions is a key factor to malaria elimination, as it enables the community to protect and sustain their own health.

Similar to other settings, such as India [18], malaria transmission was associated with poverty. Encouraging the local community to take part in the existing economical structures such as cooperatives might have a positive impact on household income, improve economic conditions, improve houses and the ability to purchase a health insurance. Cooperatives mainly operate towards financial activities. Reaching out to the community in cooperatives by programme staff may also have positive health benefits in terms of malaria elimination because preventive strategies may be tailored to a large community group at once.

Two innovative ideas came out of this research: the establishment of a rewards system and malaria clubs. A rewards system might motivate the community towards preventive behaviour but there is a need to further define the mechanisms of such system and the role of different stakeholders to be engaged. Moreover, it is deemed important to explore its implications for sustainability in the longer term. Malaria clubs have been effective in malaria elimination elsewhere through improvement in awareness on malaria among club members and the spread of information to the community. A school based program in Thailand showed success through provision of each school with teaching manuals and schoolchildren’s textbooks and held teacher training. As a result of the program, schoolchildren changed their behavior positively towards malaria prevention [19]. The school population represents more than a quarter of the total population in Ruhuha sector. Formation of similar clubs in school settings might be a great boost to the current malaria elimination programme. It is also assumed that due to students’ active engagement in malaria clubs, the knowledge and skills gained can be shared and sustained among their respective families.

The open space approach similarly to some other participatory approaches, attributes the control of discussions to the participants rather than to the organizers. The methods do consider participants as agents rather than objects, able to analyze their own problems and look for their own solutions through ownership of the knowledge and empowerment towards taking actions [8]. Open space specifically, as a result of its law of mobility, offers more flexibility for participation and contribution to various subtopics identified as priorities by participants by moving to other subgroups. Moreover, the approach does not have a limited number of participants as observed for other participatory approaches, and can be conducted among small to large groups.

## Conclusion

Results from the present research show that open space, newly deployed in health-related research, yields interesting findings as other participatory approaches and can serve as a platform to gather more information from various members of the community, to formulate measures to be taken towards elimination and create motivation for key stakeholders to be involved in such a programme, all within a short period of time. Future work will involve discussions on how agreed-upon actions will be effectively put into practice for malaria elimination in the area. Based on this, ongoing follow up to evaluate whether knowledge gained and skills learnt are shared and sustained among communities will be conducted.

### Consent

Informed consent was obtained from the participants for the publication of this report and any accompanying images.

## Abbreviations

HIV: Human immunodeficiency virus; NGOs: Non-governmental Organization; PAR: Participatory action research; PRA: Participatory rural appraisal; RRA: Rural rapid appraisal; WHO: World Health Organization.

## Competing interests

The authors declare that they have no competing interests.

## Authors’ contributions

CMI, JA, EH, FK, DM, IN and KB participated in the conception of study design. CMI, JA, EH, FK, IN, KB, DM, NK, SR, CK, MVV and BVDB performed field activities. CMI, JA, and BVDB carried out data analysis. CMI drafted the manuscript and JA, CM, LM, MVV and BVDB reviewed the paper. All authors have read and approved the final manuscript.
